# Knowledge and perceptions of primary healthcare providers towards integration of antiretroviral therapy (ART) services at departmental levels at selected health facilities Lira district, Uganda

**DOI:** 10.1186/s12913-023-09388-6

**Published:** 2023-04-24

**Authors:** Sean Steven Puleh, Emmanuel Asher Ikwara, Syliviah Namutebi, Lakeri Nakero, Godfrey Mwesiga, Rogers Isabirye, Joy Acen, Maxson Kenneth Anyolitho

**Affiliations:** 1 Department of Epidemiology and Biostatistics, Faculty of Public Health, Lira University, Lira City, Uganda; 2 Department of Community Health, Faculty of Public Health, Lira University, Lira City, Uganda; 3 Department of Psychiatry, Faculty of Medicine, Lira University, Lira City, Uganda; 4 Department of Nursing and Midwifery, Faculty of Health Sciences, Lira University, Lira University, Lira City, Uganda

**Keywords:** ART services, Readiness, Integration, Departments, Health facilities

## Abstract

**Background:**

Investigations conducted among healthcare providers to assess their knowledge and perceptions towards the integration of anti-retroviral therapy (ART) related services in Sub-Saharan Africa are limited. This study explored the knowledge and perceptions of primary healthcare providers towards the integration of ART management services at departmental levels in health facilities in Lira district.

**Methods:**

We conducted a descriptive cross-sectional survey that employed qualitative methods of data collection in four selected health facilities in Lira district between January and February 2022. The study involved in-depth interviews with key informants and focus group discussions. The study population consisted exclusively of primary healthcare providers; however, those who were not full-time employees of the participating health facilities were excluded. We used thematic content analysis.

**Results:**

A significant proportion of staff (especially those who are not directly involved in ART) still lack full knowledge of ART services integration. There was generally a positive perception, with some suggesting ART integration can minimize stigma and discrimination. The potential barriers to integration included limited knowledge and skills for providing comprehensive ART services, insufficient staffing and space, funding gaps, and inadequate drug supplies, coupled with increased workload due to enlarged clientele.

**Conclusion:**

Whereas healthcare workers are generally knowledgeable about ART integration, but their knowledge was limited to partial integration. The participants had a basic understanding of ART services being provided by different health facilities. Furthermore, participants viewed integration as critical, but it should be implemented in conjunction with ART management training. Given that respondents reported a lack of infrastructure, increased workload, and understaffing, additional investments in staff recruitment, motivation through training and incentives, and other means are needed if ART integration is to be implemented.

**Supplementary Information:**

The online version contains supplementary material available at 10.1186/s12913-023-09388-6.

## Introduction

The HIV/AIDS and COVID-19 pandemics, as well as economic and humanitarian crises, have put the global HIV response under increasing strain [1]. In Uganda, about 1.4 million people are living with HIV, including adults and children [2]. Many African countries adopted the UNAIDS 95-95-95 target of ensuring that 95% of HIV-positive people know their status, 95% of HIV-infected people receive antiretroviral therapy, and 95% of those on treatment have viral suppression [3]. The UNAIDS-focused target of near-universal 95% antiretroviral therapy (ART) access for people living with HIV (PLWHIV) by 2030 has the potential for numerous challenges [3]. Several actors have highlighted the importance of integrating ART management services at various health facility department levels, according to available evidence [4]. Those in favor of integrating HIV services with other health services argue that it has been shown to improve health and healthcare outcomes [4, 5]. Furthermore, evidence of the medical and public health benefits of integrating HIV/AIDS services appears to support it [6] through integration of HIV services with noncommunicable diseases (NCDs), TB and maternal child health [7–9]. HIV services are provided in Uganda and throughout the region by the vertically operating separately from other health system functions [10, 11] which have the potential for wastage.

Antiretroviral Therapy (ART) has been included in the Uganda National Program for Comprehensive HIV/AIDS Care and Support since 2001 [12]. According to the UNAIDS report, antiretroviral treatment coverage in Uganda is at 90% [13]. The growing number of persons living with chronic illnesses especially NCDs is on the rise in many developing nations [14]. HIV/AIDS has joined the list of chronic illnesses because many PLWHIV can now live longer with the virus because of the life-saving medicines [15]. The HIV response is evolving from a disease-specific emergency response to a chronic disease management challenge which needs to be addressed within the context of other chronic health conditions [9]. Therefore, the fast-growing number of people with other chronic diseases has considerable implications for health systems and societies [9]. This implies that substantial additional resources are needed to further scale up ART, yet funding has leveled off over recent years, increasing the need for optimizing the allocation of limited resources [16]. Moreover, with decreasing funding for vertical HIV services [10], new and innovative ways of providing care are indispensable. Although available data indicates that integrated care is highly effective and cost-effective [17], it is limited and does not provide insight into whether integration could indeed result in the hypothesized mitigation of the impact of declining HIV resources [5]. Critics have suggested that the integration of HIV services could lead to an increased volume of patients, which could potentially increase overall programme costs and resource needs [5]. Therefore, a thorough understanding of healthcare workers’ knowledge and perceptions toward ART integration at the departmental level is a novel path in the HIV care continuum. This study explored the knowledge and perceptions of primary healthcare providers towards the integration of ART management services at departmental levels in health facilities in Lira district.

## Methods

### Design and setting

The study adopted a case study design employing qualitative approaches to data collection and analysis to better understand health workers’ perceptions and attitudes toward ART integration within the mainstream healthcare system. The researchers chose this design because they wanted to gain an in-depth understanding of health workers’ opinions and perspectives on ART integration in their natural settings [18]. The study was conducted at four selected health facilities in Lira district. Lira has 16 public and 2 private health facilities providing ART services. The study was conducted at Lira Regional Referral Hospital (LRRH), Pentecostal Assemblies of God mission hospital, Ogur Health Centre IV, and Amach Health Center IV. The four health facilities were chosen due to the high number of patients on ART, and it is at these facilities that the majority of HIV/AIDS services are provided. LRRH is the major referral hospital in the Lango sub-region and a public institution administered by the Ugandan Ministry of Health (MoH). It has an out-patient department (OPD), an in-patient department (IPD), an emergency department, medicine, paediatrics, obstetrics and gynaecology, dental, laboratory, theatre, and ART departments with a total bed capacity of 254. PAG Mission Hospital is a faith-based, private, not-for-profit institution registered with the Ugandan MoH. The hospital has an OPD, IPD, ART clinic, laboratory, antenatal and postnatal care, and a young child clinic. Both Ogur and Amach are public health center IVs administered by the MoH with OPD, IPD, a theatre, maternity services and medical departments. Lira Regional Referral Hospital; has 406 health workers, and 12,275 HIV/AIDS clients; Ogur HC-IV has 2298 HIV/AIDS clients enrolled in care and 34 health workers; Amach HC-IV has 2163 HIV/AIDS clients enrolled and 28 health workers; PAG Mission hospital has 1722 HIV/AIDS clients enrolled and 83 health workers. Lira District is located about 340 km north of Kampala Uganda’s capital city. Data was collected in January and February 2022.

### Study population and procedure

This article draws on a total of 20 qualitative key informant interviews (KII) across the four facilities and 4 focus group discussions (FGD) in each of the four health facilities. When saturation was reached, the final sample size was determined. We stopped collecting data when we noticed, through reflection and critical analysis, that the next subsequent participants were providing the same type of information we required. We had planned to conduct around 20 KIIs, and this number represents the total number of KIIs planned, resulting in a 100% response rate. This could be attributed to proper mobilization combined with a good rapport, which allowed all participants to fully respond to the request to participate in the study. However, due to patient load, some of those contacted for FGDs were unable to participate. The sample size was sufficient to generate enough data to draw valid conclusions. The study population consisted exclusively of primary health care (PHC) providers. The medical profession, permanent employment, and availability and willingness to participate in the study were all inclusion criteria. As a result, the PHC providers involved in the study included medical doctors, pharmacists, clinicians, nurses, midwives, laboratory technicians, and counselors. The rationale for this is that the participants are likely to be knowledgeable and well-informed about the ART services available within their units and health facilities. Exclusion criteria include those in non-medical professions, those not working full-time, those who are not available, and those who are unwilling to participate in the study. The main reason for this exclusion was that those in non-medical professions would not be able to fully comprehend the topic, and those who are not in full-time employment are frequently mobile and may not have had a proper grounding in their work environments.

### Sampling criteria

We purposefully selected the four health facilities in the greater Lira district considering their number of departments. The four facilities include two health center IV (HCIV), one Private not for profit (PNFP), and one regional referral hospital (RRH). They were selected because of the number of departments. Health center IIIs were not considered because of limited departments. We also purposively recruited PHC providers for key informant interviews and focus group discussions depending on their roles within the departmental units and work experience (at least 6 months at the current health facility). All participants were eligible for both data collection methods.

### Data collection tools and procedures

A semi-structured topic guide was designed to elicit information on primary health workers’ knowledge and perception of the integration of ART services and whether it can produce long-term health outcomes. The study tools for interviews were modified from the WHO reproductive health readiness assessment-hexagon tool [19]. According to this tool, the domains of readiness assessment include needs, fit, resources, capacity, readiness, and evidence. A modified tool was designed by the research team to fit the context of the study setting (Additional file 1). The study tools were pretested on two and five PHC workers at a non-participating health facility for KIIs and FGDs, respectively, before actual data collection, and the information obtained was used to improve the tools. Three research assistants with social science backgrounds were recruited and trained on quality data before data collection. Face-to-face interviews were conducted in English among 20 key informants and 35 focus group discussants. The KIIs and FGDs were used in this study because they allowed participants to express themselves freely in their natural settings rather than relying on pre-programmed responses. The KIIs and FGDs were conducted privately, at locations within the health facility preferred by the interviewers where there would be no interference. We structured focus group discussions consisting of multi-disciplinary PHC workers, and the interviews were conducted by experienced interviewers who made sure that everyone was involved. We obtained written informed consent from all participants before they were interviewed. Key informant interviews were designed to take approximately 20 min to complete to avoid interfering with the patient flow. All interviews were audio-recorded. Written informed consent was sought from all respondents before interviews were conducted. The KII took an average of 20 min, and the FGDs averaged about an hour. Using a prepared FGD Guide one moderator asked participants a series of open-ended and probing questions. The audio recorder recorded the discussions while also taking notes and capturing facial and body expressions. To ensure privacy, the discussions were held in meeting rooms. Data collection took place between January and February 2022.

### Data management and analysis

**Table 1:** Thematic Analysis Table

Main Themes	Sub-theme 1	Sub-theme 2
Knowledge of ART services	• Provision of ARV drugs	
	• A place for service provision	
	• A range of services	
	• Inadequate knowledge	Lack of experience
		Not being deployed at the ART unit
Knowledge of ART services integration	Correct knowledge	Mixing of clients across departments
		Integrating HIV service with other services
		Beneficial service delivery
		ART service provision to different departments
		Outreach oriented integration
	Incorrect knowledge	Providing a range of HIV services at the same time
		Screening HIV patients for many diseases
Perceptions regarding ART services integration	Positive perceptions	Information Sharing
		Time-saving
		Increased confidentiality
		Reduced stigma
		Easy drug accessibility
		Increased number of clients
	Negative perceptions	Increased stigma
		Discomfort in drug dispensation
		Limited attention to clients
		Knowledge gap by some health workers
		Risk of contracting other diseases
		Increased workload and limited space
Perceived facilitators to ART services integration	Good infrastructures	
	Recruitment and proper training	
	Adequate financial facilitation	
	Teamwork	
Perceived barriers to ART services integration	Knowledge gap	
	Lack of trained personnel	
	Work overload	
	Lack of testing kits	
	Inadequate infrastructure	
	Lack of space	

Thematic content analysis was used to analyze the data. All the KIIs and FGDs, and all audio recordings were reviewed and stored on hard disc drives only accessible to the study team. The dataset was then transcribed verbatim by the research assistants. All transcripts were reviewed for completeness and serially numbered. The transcripts were then uploaded onto NVIVO version 12 software for onward analysis. Transcripts were read and re-read by the research team to familiarize themselves with the data. Next, three transcripts, two from KII and one from FGD, were selected and read again, along with the study guides, to come up with a code book detailing various initial codes that emerged from participants’ responses and the tools. Later all the remaining transcripts were read one by one, to guide further coding. Any new and emerging codes were identified and added to the existing ones. The actual codes were chosen after a consensus among the team. Themes and subthemes were later developed from the codes, and thereafter, descriptive interpretation was supported by verbatim quotes (Table 1).

## Results

We set out to assess healthcare workers’ level of knowledge and perceptions regarding the integration of ART services into mainstream departments, units, and clinics. Data was collected from 54 participants, including 20 KIIs and 34 FGD participants.

### The socio-demographic characteristics of the participant

In this study majority 57% (31/54) of the participants were female, and nurses had the most numbers 30% (16/54) (Table 2).

**Table 2:** The socio-demographic characteristics of the participant

Variable	Frequency (n)	Percentage (%)
**Sex**		
Females	31	57
Males	23	43
**Age**		
20–25	09	17
26–30	14	26
31–35	12	22
36–40	09	17
41–45	05	09
**>** 45	05	09
**Cadre**		
Nurses	16	30
Midwives	7	13
Counsellors	6	11
Lab Technician	11	21
Clinical Officer	6	23
Medical Officer	1	2
**Health Facility**		
Lira Regional Referral Hospital	16	30
PAG Mission Hospital	16	30
Ogur HC-IV	10	18
Amach HC-IV	12	22

### Knowledge of healthcare workers regarding the integration of ART services

The first objective of the study was to assess healthcare workers’ level of knowledge regarding ART services integration. Healthcare workers expressed varied knowledge regarding ART services and their integration. ART services were perceived to refer to services given to prolong the lives of people, taking ARV drugs to help in the prevention of other diseases and the provision of counselling to those infected and affected. Furthermore, knowledge of integration was categorized into correct and incorrect depending on whether or not they were able to give a clear description of integration.

Some participants saw ART as the provision of ARV drugs to those infected with the disease. They went further to state that the drug is not a cure but helps boost a person’s immunity thereby prolonging their life. Below are voice excerpts from some of the study participants:

*“My knowledge about ART is that ARV is given to someone who has tested (HIV) positive and we tell them that this drug does not cure HIV but it just boosts your immunity to fight other diseases, so we have to encourage them to take the drug since it prolongs their life” (FGD Participant 3 Lira RRH)*

Other participants however explained ART as a place where HIV services are provided. This was mainly expressed by the KII participants as seen in the below quote;

*“This is a place where we have clients who are HIV positive. When you are put on ART care, we take it that you are now on medications, that’s what I know about ART” (KII 20 Ogur HCIV)*

Knowledge of ART services were mostly expressed in terms of the big thing. That is, participants were able to give a comprehensive account of what ART services entail. They said ART services involve a whole process, beginning from the detection of the virus through testing, then counselling the clients, initiating them into the system, and afterwards enrolling them. Some of the participants said that...

*“ART is a big thing; I would say it starts from the detection of HIV in clients who come here for routine services. Once they are detected they go through counselling, and the journey starts like that. Then also comes the issue of ART initiation or enrolment for care, and then follow-up. When they want to see whether they are responding to treatment or not.” (KII 14 PAG Mission hospital)*

On the other hand, some participants reported inadequate experience with ART services. They attributed it to a lack of experience as a result of not working at the ART units/departments. This was revealed by a failure to understand what it is, and the services provided.

*“I have never worked in the ART department, but I see them and I handle them once they have been sent to this department.” (KII 13 Amach HCIV)*

Some participants stated that integration of ART services means the mixing of those clients across the different departments at the hospital. They reported that clients can go and be served at any of the departments within the facility. Such participants justified integration in terms of minimizing stigma and discrimination. One of them said thus;

*“What I know, like the way I was trying to reason is like everyone will be mixed, those in ART can go to OPD, they get the service together...........whereby they see these people together not segregating that this is from ART or from what” (KII 20 Ogur HCIV).*

Meanwhile, other participants understood ART service integration as the provision of HIV services across the different departments. To such participants, integration is about service and not so much about stigma or discrimination.

*“I think it is just all about integrating ART with other services, OPD services when you are seeing OPD patient, then you offer Art services at the same time in the same house just like at the dispensary, they bring ARV, they put them there for clients to pick.” (KII 3 Amach HCIV)*

Some participants interpreted “integration of ART services” to mean providing multiple services to clients at the same time. They looked at integration as going for outreach services. A key informant from the PAG mission hospital said thus;

*“Aaa recently I have seen staff move out of the facility, so that they do comprehensive services out there. Like there is moonlight testing where you go to selected villages, and then do HIV testing and counselling, and TB services” (KII 14 PAG Mission hospital)*

The data show that, in general, the participants had some ideas about what integration means in terms of ART services. However, there were a few that could not clearly explain the concept.

### Healthcare workers’ perceptions regarding the integration of ART services

The perceptions of healthcare workers regarding the integration of ART services into mainstream healthcare systems and structures were explored. In this case, integration was categorized into positive, negative, both positive and negative, and undecided opinions. Furthermore, we asked participants to give reasons for their views and opinions.

The dominant view among participants was that integration is a good model of care and should be supported by everyone. The views were held by participants from different facilities and different units/departments and were shared by both the FGD and KII participants. Participants observed that integration would bring advantages for information sharing, save time, promote confidentiality, reduce stigma, reduce workload, and make drug accessibility easier than before. Furthermore, according to participants, reduced stigma would lead to an increase in the number of clients coming for ART services. This group of participants had the following to say;

*“Yes, this because it saves time for both patients and health workers since both of them will not be moving from one department to another, makes it easy to access the drugs, and also reduces the workload.” (KII 15 PAG Mission hospital)*

Furthermore, participants argued that ART service integration reduces stigma. A few other participants agreed that ART integration could reduce stigma if the drugs were not easily identifiable. They stated the following:

***“****It is another way of the fighting stigma of those who may feel self-stigmatized. Bringing them together will make them not fear to come.” (FGD Participant 2 PAG Mission hospital).*

Some of the participants said that ART service integration is good because it reduces the volume of work, especially the ones that used to be provided at the ART clinics. Integration, according to the participants, implies that some of the services will be provided by staff in other departments. Furthermore, they reported that it makes drugs easily accessible to clients, as they do not have to go all the way to the ART clinic if they are from another department.

*“Integration of ART clinic will also reduce on the tendency of workload among staff. It will provide easy access to drugs since everywhere you want to get assistant; you will be in a position to get.” (FGD Participant 2 Lira Regional Referral hospital)*

Generally, participants expressed a strong view that proper training would enable integration to be successful. These in-service training could be organized by those who have had the training earlier, as continuing medical education (CME), and in the form of capacity building. Below is what some of the participants had to say:

*“Training the staff on ART management services, recruitment of more knowledgeable staff, providing of more drugs, and sensitization of staff on ART related issues and action of staff and clients” (KII 15 PAG Mission hospital)*

Participants reported that, together with more training, there should also be the recruitment of more staff. This, according to participants, would help address the barrier of work overload. Participants argued that

*“More staff are to be recruited in the health facility in the entire department. There is need for more manpower to help the overwhelming clients at health facility”* (FGD Participant 1 Lira Regional Referral hospital)

Other benefits mentioned by the participants in support of integration include increased drug supplies to handle the issue of drug shortages; improved use of infrastructure to address the problem of limited space.

*“Drugs will be supplied mainly for ART services and there will be proper use of the available space” (FGD-2, Lira regional referral Hospital).*

Financial motivation and teamwork, among others, were also highlighted by participants as benefits of integration. Staff will be motivated to handle the increased clients and workload.

*“I think it is just a matter of teamwork; ART needs team work because for that ART to exist, the Lab should be in contact with them, the TB department and so on, so we work as a team.” (KII-1Ogur HCIV)*

*“We have a good number of health workers in the facility who are healthy and energetic and knowledgeable who love teamwork because all of us love supporting one another, so in case of anything everybody can come in to give a hand. And working in a team can have some good benefits”* (FGD − 2 LRRH)

On the other hand, findings from the data also reveal that a few participants thought that integration is not a good model of care. They gave reasons such as increased stigma towards HIV-positive clients, discomfort with the way drugs will be dispensed, limited attention to clients, a lack of experience among healthcare workers, drug shortage, lack of testing kits, and distortion of schedules, among others.

According to some of the participants, integration is likely to increase stigma due to the lack of privacy when providing the service. Some of the participants had this to say:

*“Because it will increase stigma, among the clients, since those clients use the containers when picking their drugs which make a lot of noise. So, everyone will just get to know that they are taking ARV” (FGD Participant 3 Lira Regional Referral Hospital)*

Still, to other participants, ART integration looks beneficial to clients but not to staff. This is because of too much work, and limited space among others. They stated as follows;

*First of all, space will not be enough because we are handling many clients within the same space, so that is the challenge. It requires a lot of rearrangement, structural adjustments, and staffing, it needs a lot of adjustments to be made if that idea is to work.” (KII 2 Ogur HCIV)*

*“Just like you are seeing the space is very small to accommodate all the clients that have come to seek their services and also the insufficient drugs in the facility”.* (FGD-10 Lira Regional Referral Hospital)

Participants reasoned that a lack of trained personnel would make it difficult to provide the required services. Below is what some of them had to say;

*“There are inadequately trained staffs in ART management, and poor facilitation of the health workers” (KII 9 Amach HCIV)*

Regarding limited space, participants looked at many clients coming to different sections that were originally not planned for them. This was more pronounced at the lower-level facilities, even though the same was mentioned for the regional referrals and by both FGD and KII participants. Some of them said thus;

*“The rooms are congested because there are those who have come for family planning, drugs refill, and deliveries, yet all these different services have to be provided in the same place. Therefore, everyone is mixed up.” (FGD participant 4 Amach HCIV)*

Also, work overload was viewed by the participants as another possible factor that would hinder integration. This point was emphasized by nearly all participants across different categories and across different facilities, highlighting its importance.

*“Too much workload, this is because single staff have to handle the patients from the different department, for example malaria versus HIV, TB vs HIV, and others” (KII 15 PAG Mission hospital)*

## Discussion

This study aimed to assess healthcare workers’ levels of knowledge and perceptions regarding the integration of ART services. The study discovered that the participants at the four facilities had a disproportionate amount of knowledge and experience with ART service integration. Furthermore, as discussed, they had differing perceptions of their perspectives on ART integration.

According to our findings, most healthcare workers had adequate knowledge of the ART services offered at the health facilities. Healthcare workers expressed varied knowledge regarding ART services and their integration. The variation in knowledge could be attributed to the difference in the categories of health care providers who were interviewed and the different in-service trainings they underwent. Our finding is similar to evidence from the elsewhere which presented variation in the level of knowledge of HIV services by the care providers [20]. The lack of knowledge if not addressed could lead to stigmatization by the healthcare workers. Other studies have found that a lack of knowledge about HIV and AIDS among the health care workers is a predictor of stigmatization of PLWHIV [21]. Therefore, future programming should use an inclusive approach to the training of staff at health facilities and not only those at the ART clinics.

Regarding knowledge of the integration of ART services, our finding reveals some good understanding of integration by participants. Staff at various units were able to explain and describe the integration of ART services into different departments and units. This good knowledge could be attributed to the experience PHC providers had with some aspects of partial integration such as family planning, TB, and ANC. This current study is supported by findings from other studies which revealed that staff were not only knowledgeable about integration but were already implementing it within other PHC components [22], both at the national and at sub-Saharan level [5]. It should be noted, however, that program implementation does not always imply knowledge of it. This could explain why, in our current study, some participants stated that they were unaware of it, even though they claimed to provide ART services. Therefore, before implementing integration, we recommend staff training which is in line with a study conducted in Tanzania [23]. Assessing provider knowledge about integrating ART into the department may have been more effective and clearer after service integration [24].

According to the current study, there is a generally positive perception of ART service integration into mainstream PHC. Integration was reported to be not only desirable, but also feasible and necessary for improving the health outcomes of HIV-positive clients and society as a whole. Participants gave a variety of reasons for their positive perception, including the benefits of information sharing, time savings, stigma reduction, easy access to services, reduced workload, and, ultimately, an increase in the number of clients accessing ART services. The findings are consistent with evidence from Namibia, which found that integrating HIV/SRH services improved ease of access, reduced stigma, maternal healthcare performance, and nurse performance while decreasing time spent in the health facility without sacrificing care or service uptake [25]. Other studies, on the other hand, have found that integrating ART services into other departments and units is necessary for staff productivity and overall improvement in health service provision [26, 27]. Indeed, with integration comes a more cost-effective and efficient approach to health service delivery [28].

Our study also found that ART integration within the mainstream PHC would come with proper staff recruitment, financial motivation and teamwork, training, and increased drug supplies, among others. This could partly be explained by the funding opportunities available for the HIV programming. In addition, some aspects of ART were successfully integrated with some aspects of healthcare such as family planning and ANC among others. There is therefore, no doubt that full integration of ART into different departments could yield massive gains in the delivery of healthcare services. Our study is supported by a previous study in Tanzania, which also found that proper staff training is likely to facilitate successful integration, whereas the opposite is true [23]. This is possiblly due to the similarity of the contexts in which the two studies were carried out.

However, this study discovered that a subset of participants still believed integration was a poor model of care and thus unnecessary. Such participants reasoned that integration would increase staff workload and create stigma among clients. This is most likely because, in general, health care providers face heavy caseloads as a result of understaffing at the majority of health facilities in resource-limited settings. This could imply that having more clients for various departments means having more work for the same staff at the same time. Similar to the findings of this study, have also supported this finding, stating that the integration of ART services may result in increased workload for the staff [26]. More education about the benefits of ART integration is likely to change such negative perceptions.

Our current study found that inadequate knowledge and skills, lack of trained healthcare personnel, and workload among others are some of the factors that could hinder successful ART integration. Since ART integration is still a new concept, it is possible that some people may not fully comprehend it and therefore may not appreciate its importance. However, with continuous training, resource allocation and other support, such gaps could be bridged. Previous studies have also indicated that a lack of trained staff, supply chain challenges, funding gaps, and stigma, among others, can act as impediments to the implementation of integrated ART services [29, 30].

Finally, this study provides some insights into primary healthcare workers’ knowledge and perceptions of ART service integration at the departmental level. Our study, on the other hand, had some limitations. For example, the cadres chosen for the focus group discussion were diverse, which could have influenced the responses. We did, however, use experienced moderators to ensure that everyone contributed to the topic of discussion. Furthermore, our study was conducted in a single district, focusing on a regional referral hospital, one PNFP hospital, and two level four health facilities, which may have an impact on the generalizability of our findings. Furthermore, we were unable to assess knowledge differences among health workers based on cadre type.

## Conclusions and recommendations

In this study, whereas healthcare workers are generally knowledgeable about ART services being provided by different health facilities and have some ideas about integration, their knowledge is only limited to partial integration. Furthermore, although there is generally a positive perception of ART service integration, in the study setting we discovered that some healthcare workers are still skeptical about ART integration. This current study recommends that adequate and proper training in ART services be given to all staff in the departments to equip them with the skills and knowledge to handle ART-related services coupled with adequate financial motivation. In addition, the study recommends the integration of staff in the ART clinics and, where necessary, recruiting more staff to address the issue of increased workload and demand for services. Furthermore, future studies could assess the differences in knowledge among health workers based on the type of cadre.

## Electronic supplementary material

Below is the link to the electronic supplementary material.

Supplementary Material 1

## Data Availability

The datasets used and/or analyzed during the current study contains data used in another publication and are available from the corresponding author upon reasonable request.

## List of Abbreviations

ANC: Antenatal care
ART: Anti-retroviral therapy
FGD: Focus Group Discussion
GUREC: Gulu University Research and Ethics Committee
HC: Health center
IPD: In-patient department
KII: Key informant interviews
LRRH: Lira Regional Referral Hospital
NCD: Non-communicable diseases
OPD: Out-patient department
PAG: Pentecostal Assemblies of God
PHC: Primary health care
PLWHIV: Person’s living with HIV/AIDS
PNFP: Private not for profit
SSA: Sub-Saharan Africa
TB: Tuberculosis
