# Supplementary material for: Knowledge and perceptions of primary healthcare providers towards integration of antiretroviral therapy (ART) services at departmental levels at selected health facilities Lira district, Uganda
Source: BMC Health Serv Res. 2023 Apr 24;23:394. doi: 10.1186/s12913-023-09388-6 (PMC10123554; doi:10.1186/s12913-023-09388-6)
Supplement: Supplementary file 1 — Supplementary Material 1 [file 12913_2023_9388_MOESM1_ESM.docx]

## Additional file 1: Key informant interview guide

**Project Title:** Readiness of primary healthcare facilities towards integration of ART services at departmental level in Lira district.

**A. Perceptions of primary healthcare provider towards the integration of ART services at departmental levels.**

1. What are your main duties and responsibilities in this facility?
2. What is going on well within the duties and responsibilities you are engaged in this facilities?
3. What is not going on well within the duties and responsibilities you are engaged in this facilities? (Mention at least six areas).
4. What is your knowledge about ART?
5. What is your opinion about ART services integration into the departments at the health facility? (Probe; how and why)
6. Have you ever heard about integration of ART management services?
7. What do you know about ART management services integration?
8. What are some of the hindrance to ART management services integration at departmental level that you anticipate?
9. In your opinion, what do you think will enable the integration to be a success? (Probe; how and why)

**B. Attitudes of primary healthcare providers towards the integration of ART services at departmental levels of four health facilities in Lira district**.

1. What is your opinion about ART services being integrated at departmental levels in this facility?
2. What do you think is required to integrate ART services at departmental levels in this facility?
3. How do you feel about having ART services integrated at departmental levels in this facility?

**C. Readiness of Primary Healthcare Facilities towards the integration of ART services at departmental levels in Lira district.**

1. **Preparedness:** What is your opinion about the preparedness of this facility on the integration of ART services at departmental levels?
2. **Fitness:** What is your opinion about integration of ART services at departmental levels fitting the current guidelines, protocols and initiatives of this facility?
3. **Resources:** What is your opinion about the suitability of the available resources at this facility concerning integration of ART services at departmental levels?
4. **Needs:** What is your opinion about the suitability of the available systems at this facility concerning integration of ART services at departmental levels?
5. **Capacity:** What is your opinion on the qualification capacities of human resources of this facility as far as the integration of ART services at departmental levels is concerned?
6. **Evidence:** In your opinion, what are some of the expected outcomes of integration of ART services at departmental levels?

**D. Is there any other thing you would love to share with me concerning ART management services integration at departmental level?**

## Additional file 2: Focus Group Discussion guide

**Project Title:** Readiness of primary healthcare facilities towards integration of ART services at departmental level in Lira district.

**A. Perceptions of primary healthcare provider towards the integration of ART services at departmental levels.**

1. What are the main HIV services provided in this facility?
2. What is going on well with the HIV related services in this facilities?
3. What is not going on well with the HIV related services in this facilities? (Mention at least six areas).
4. What is your knowledge about ART?
5. What is your opinion about ART services integration into the departments at the health facility? (Probe; how and why)
6. Have you ever heard about integration of ART management services?
7. What do you know about ART management services integration?
8. What hindrances to ART management services integration at departmental level do you anticipate?
9. In your opinion, what do you think will enable the integration to be a success? (Probe; how and why)

**B. Attitudes of primary healthcare providers towards the integration of ART services at departmental levels of four health facilities in Lira district**.

1. What is your opinion about ART services being integrated at departmental levels in this facility?
2. What do you think is required to integrate ART services at departmental levels in this facility?
3. How do you feel having ART services integrated at departmental levels in this facility?

**C. Readiness of Primary Healthcare Facilities towards the integration of ART services at departmental levels in Lira district.**

1. **Preparedness:** What is your opinion about the preparedness of this facility on the integration of ART services at departmental levels? (Probe for preparedness)
2. **Fitness:** What is your opinion about integration of ART services at departmental levels fitting the current guidelines, protocols and initiatives of this facility?

- **Resources:** What is your opinion about the suitability of the available resources at this facility concerning integration of ART services at departmental levels? (Probe for resources available)

1. **Needs:** What is your opinion about the suitability of the available systems at this facility concerning integration of ART services at departmental levels? (Probe the existing systems, data and evaluations)
2. **Capacity:** What is your opinion on the qualification capacities of human resources of this facility as far as the integration of ART services at departmental levels is concerned? (Probe for capacity)

- **Evidence:** In your opinion, what are some of the expected outcomes of integration of ART services at departmental levels? **(**Probe for the anticipated outcomes)

**D. Is there any other thing you would love to share with me concerning ART management services integration at departmental level?**
